# The Link between Right and Left Ventricular Systolic Performance at Rest and after Stress: Insights Into the Mechanism

**DOI:** 10.4021/cr291w

**Published:** 2013-10-15

**Authors:** Dawod Sharif, Amal Sharif-Rasslan, Camilia Shahla, Amin Khalil, Uri Rosenschein, Majed Odeh

**Affiliations:** a Department of Cardiology, Bnai Zion Medical Center, Haifa, Israel; b Technion - Israel Institute of Technology, Haifa, Israel; c Department of Internal Medicine A, Bnai Zion Medical Center, Haifa, Israel

**Keywords:** Echo-Doppler, Systolic function, Contraction reserve, Dobuatmine

## Abstract

**Background:**

Right ventricular (RV) systolic performance is more difficult for evaluation compared to the left ventricle (LV). Despite differences in structure, RV myocardial fibers are in continuity with those of LV. The aim is assessment of the effects of LV wall motion abnormalities (WMA) on RV systolic function at rest and after stress.

**Methods:**

Fifty nine subjects, 15 with LV-WMA underwent dobuatmine stress echocardiography (DSE) studies using the usual protocol. Measurement of tricuspid annular plane systolic excursion (TAPSE), velocity (TASV), mitral annular plane systolic excursion (MAPSE) and velocity (MASV), were performed before and immediately after DSE studies.

**Results:**

TAPSE was lower, in those with LV-WMA than in those without, both at rest 20.5 ± 4.8 mm versus 24.9 ± 4.7 mm, P = 0.015 and after DSE studies, 21.5 ± 5.6 mm versus 27.65 ± 5.7 mm, P = 0.005. DSE studies did not change TAPSE significantly in the presence of LV-WMA. TASV at rest in those with LV-WMA was 16.5 ± 2.7 cm/sec and similar to that in those without, 17.6 ± 3 cm/sec. In both groups the velocity increased after DSE studies, 23.25 ± 7.5 cm/sec, P = 0.01 with LV-WMA, and 27.5 ± 6 cm/sec, P = 0.0005, without LV-WMA. Despite similar TASV at rest, the TAPSE/TASV ratio, indicating duration of shortening, was lower (124 ± 21 msec) in subjects with of LV-WMA, than in those without (145 ± 27 msec), P = 0.0065, implying increased after load for RV longitudinal shortening in the presence of LV-WMA.

**Conclusions:**

TAPSE is lower at rest and after DSE studies in subjects with LV-WMA than in subjects without; however, DSE studies increase TPASE only in the absence of LV-WMA. TASV increases after DSE studies and is similar at rest in both groups with or without LV-WMA. It seems that LV-WMA increases after load to RV longitudinal motion.

## Introduction

Evaluation of left ventricle (LV) systolic function by trans-thoracic echocardiography is standardized and widely applied. Previously, the dynamics of changes in longitudinal LV systolic function induced by stress have been reported [1]. On the other hand, right ventricular (RV) geometry is complex and as a result, it is often not adequately evaluated. When attempted, frequently, RV systolic function is evaluated by the eye-balling method. Objective evaluation of systolic RV function may be achieved by using the systolic percent area change [2], or more recently, by using three-dimensional echocardiography [3].

Longitudinal systolic performance abnormalities precede LV systolic radial abnormalities [4]. Tricuspid annular plane systolic excursion (TAPSE) may be applied to estimate RV systolic performance [2, 5, 6]. In addition, RV muscle fibers are linked to those of LV [7, 8]. Therefore, the present study was performed in order to evaluate the effects of LV wall motion abnormality (WMA) on RV systolic function and reserve, and to explore the possible mechanisms of the interaction between LV and RV.

## Methods

### Population

Fifty eight patients, 29 women, age 60.3 ± 13.4 years, were prospectively evaluated for the presence of coronary artery disease. Fifteen of the subjects had LV-WMA at rest. Patients with pulmonary disease, right heart disease, valvular heart disease, atrial fibrillation or other rhythm disturbances and acute coronary syndrome within 3 months were not included. All underwent dobutamine stress echocardiography (DSE) studies using the usual protocol and measurement of RV and LV systolic function before and after DSE studies.

### Dobutamine stress echocardiography

The protocol of dobutamine infusion consisted of 3 minute stages for each dose, starting with 5 µg/kg/min and increasing to 10, 20, 30 and 40 µg/kg/min. If end-points did not occur or 85% of the age adjusted heart rate was not achieved, 0.25 mg atropine was injected every 2 minutes up to 1 mg or until the target heart rate was achieved. Blood pressure and 12 lead electrocardiograms were recorded at rest and throughout the DSE study. Horizontal or down-sloping > 1 mm ST-segment depression at 0.06 sec after the J point were considered as evidence for myocardial ischemia.

### Image acquisition

Images were obtained while the patients in the left lateral decubitus position. A standard commercial Siemens, Acuson Sequoia echocardiography system, California, equipped with 3.5 MHZ transducers was used. All patients had complete Doppler echocardiographic studies before DSE. Parasternal long axis and short axis, as well as, apical 4-chamber and 2-chamber views were recorded at rest, low dose dobutamine infusion, peak exercise and in the recovery period. Digital images were stored on magneto-optic discs for later off-line analysis. In addition super VHS videotape recordings were performed throughout the studies.

### Dobutamine stress echocardiographic analysis

Segmental LV wall motion analysis was performed using 16-segment model [7]. Regional wall motion and segmental score was estimated as normal = 1, hypokinetic = 2, akinetic = 3 or dyskinetic = 4. New or worsening segmental wall motion was considered as ischemic response. Ischemic response (I) was identified when wall motion decreased by at least 1 grade in 2 adjacent segments or wall motion decreased by at least 2 grades in 1 segment, otherwise no ischemia, or normal response (N) was diagnosed. Wall motion score index (WMSI) was calculated as: WMSI = (Sum of scores of 16 segments)/16.

### Longitudinal RV and LV systolic function

Measurements of TAPSE (mm) using M-mode echocardiography at the lateral border of the tricuspid annulus from apical four chamber view, and TASV (cm/sec) from the same approach and site using pulsed-wave tissue Doppler imaging were performed. Mitral annular plane systolic excursion (MAPSE) from M-mode echocardiography (mm) of the mitral annulus at the lateral and septal borders, and mitral annular systolic velocity (MASV) from the same sites using pulsed-wave tissue Doppler imaging (cm/sec) were performed. Ratios of TAPSE/MAPSE and TASV/MASV were calculated. Measurements and calculations were performed at rest and immediately after DSE.

### Statistical analysis

Mean values and standard deviation of TAPSE, MAPSE, TASV, MASV and ratios were calculated for all subjects. Student t-test assuming unequal variances, was performed; P < 0.05 was considered significant. Comparison was performed between subjects with and those without LV-WMA. Comparisons between RV and LV findings were performed using student t-test.

## Results

All subjects underwent DSE studies safely and uneventfully. In subjects without LV-WMA, heart rate increased from 70.7 ± 10 bpm to 138 ± 11.8 bpm, P < 0.00001, and in subjects with LV-WMA increased from 74.2 ± 17.6 bpm to 130.9 ± 15.6 bpm, P < 0.00001. Heart rate was similar between groups both at rest and after DSE studies. Systolic blood pressure increased from 138 ± 7 mmHg to 162 ± 9 mmHg, P < 0.0001, in all subjects without significant inter-group difference.

### Left ventricular wall motion

In 26 subjects WMSI was 1 at rest and did not change after DSE studies, while in 18 subjects WMSI was 1 at rest and increased to 1.12 ± 0.14 after stress. In 15 subjects WMSI at rest was 1.36 ± 0.34 and increased to 1.47 ± 0.32 after DSE studies.

### Tricuspid annular systolic excursion (TAPSE) and velocity (TASV)

TAPSE and TASV were similar at rest and after DSE in normal subjects and in those with LV-WMA only after DSE (Table 1). Therefore, further analyses and comparisons were performed between subjects with LV-WMA at rest and those without resting LV-WMA.

**Table 1:** RV Longitudinal Systolic Function Parameters at Rest and After DSE Studies in Normal Subjects With and in Those With LV-WMA Only After DSE

		REST	DSE	P
TAPSE (mm)	WMA after DSE	24.04 ± 4.2	27.2 ± 5.9	0.13
	NO-WMA	24.9 ± 4.8	27.7 ± 6.2	0.062
	P	0.556	0.818	
TASV (cm/sec)	WMA after DSE	16.9 ± 4.1	26.4 ± 8.3	0.0007
	NO-WMA	17.6 ± 3.4	27.5 ± 7.2	1.37 × 10^-11^
	P	0.59	0.64	
TAPSE/TASV(msec)	WMA after DSE	147.6 ± 33.6	108 ± 23.3	0.0073
	NO-WMA	143.4 ± 29.3	102.6 ± 22.5	3.3 × 10^-7^
	P	0.82	0.105	
HR (bpm)	WMA after DSE	70.3 ± 8.8	135.5 ± 14.3	5.2 × 10^-15^
	NO-WMA	70.7 ± 9.96	139.6 ± 9.9	3.08 × 10^-44^
	P	0.81	0.308	

In subjects with LV-WMA at rest, TAPSE was lower than in those without resting LV-WMA, both at rest and after DSE, indicating lower RV systolic function (Table 2). While in subjects with resting LV-WMA, no significant change in TAPSE was found after DSE; in those without resting LV-WMA, TAPSE increased significantly after DSE, implying good RV systolic function reserve. Contrary to TAPSE, TASV was similar in both groups at rest and after DSE studies (Table 2). TASV increased during DSE in both groups with or without resting LV-WMA, but more significantly in those without LV-WMA at rest. Despite similar heart rate in both groups (Table 2), effective average tricuspid systolic displacement time as measured by TAPSE/TASV was larger at rest, but not after DSE studies, in those without LV-WMA at rest (Table 2). After DSE studies the increase in TASV was associated with shortening of TAPSE/TASV, more so in those without resting LV-WMA (Table 2).

**Table 2:** RV Longitudinal Systolic Function Parameters at Rest and After DSE Studies in Subjects With and Without LV-WMA at Rest

		REST	DSE	P
TAPSE (mm)	WMA	20.5 ± 4.8	21.5 ± 5.6	0.3
	NO-WMA	24.5 ± 4.5	27.5 ± 5.7	0.006
	P	0.0088	0.002	
TASV (cm/sec)	WMA	16.5 ± 2.7	23.3 ± 7.5	0.0057
	NO-WMA	17.3 ± 3.4	27.1 ± 6.8	1.37 × 10^-11^
	P	0.2	0.06	
TAPSE/TASV (msec)	WMA	124 ± 21	96 ± 19	0.0019
	NO-WMA	145 ± 27	105 ± 22	5.01 × 10^-10^
	P	0.0065	0.105	
HR (bpm)	WMA	74.2 ± 17.6	130.9 ± 15.6	4.35 × 10^-10^
	NO-WMA	70.7 ± 9.96	138 ± 11.9	3.08 × 10^-44^
	P	0.48	0.123	

### Relation of RV and LV systolic function and reserve

RV longitudinal systolic function as measured by TAPSE was coordinated with LV septal and lateral excursions both at rest and after DSE studies. Thus, TAPSE/MAPSE did not change after DSE studies (Table 3). Moreover, the coordination between RV and LV systolic displacements was maintained in subjects with or without LV-WMA (Table 3).

**Table 3:** Relationship Between Longitudinal RV and LV Ventricular Systolic Function Parameters at Rest and After DSE Studies in Subjects With and Without LV-WMA at Rest

			REST	DSE	P
TAPSE/MAPSE	IVS	WMA	2.27 ± 1.02	2.08 ± 0.87	0.65
		NO-WMA	1.87 ± 0.36	2.0 ± 0.5	0.25
		P	0.247	0.77	
	LATERAL	WMA	1.62 ± 0.46	1.64 ± 0.55	0.93
		NO-WMA	1.62 ± 0.39	1.72 ± 0.5	0.28
		P	0.95	0.66	
TASV/MASV	IVS	WMA	1.68 ± 0.74	1.81 ± 0.46	0.62
		NO-WMA	1.61 ± 0.34	1.5 ± 0.3	0.155
		P	0.76	0.058	
	LATERAL	WMA	1.77 ± 0.93	1.82 ± 0.58	0.89
		NO-WMA	1.52 ± 0.43	1.58 ± 0.47	0.53
		P	0.38	0.21	

In addition, TASV was coordinated with that of LV. Presence or absence of LV-WMA did not affect this inter-ventricular coordination both at rest and after DSE studies (Table 3).

As depicted in Table 3, RV longitudinal systolic displacement and velocities were higher than those of LV. However, lateral TAPSE, despite being larger than lateral MAPSE, their association was closer than that with septal MAPSE (Table 4). These septal-lateral differences were evident at rest in those with or without LV-WMA; however, after DSE this difference disappeared in the presence of LV-WMA (Table 4). Rate of change of longitudinal systolic displacements-TASV and MASV did not show these septal-lateral LV differences in relation to RV, both at rest and after DSE studies, irrespective of presence or absence of LV-WMA (Table 4).

**Table 4:** Comparison of Ratios of RV to LV Ventricular Septal and Lateral Systolic Function Parameters in Subjects With and Without LV-WMA at Rest

		P (IVS:LATERAL)	
		Rest	DSE
TAPSE/MAPSE	WMA	0.0753 (0.038)	0.172 (0.086)
	NO-WMA	0.0016 (0.0008)	0.0153 (0.0076)
TASV/MASV	WMA	0.78 (0.39)	0.96 (0.48)
	NO-WMA	0.33 (0.16)	0.36 (0.18)

P value statistics two tailed and one tailed (in brackets) student test.

## Discussion

In this study it was shown that RV systolic function at rest and after DSE as measured by TAPSE was lower in those with LV-WMA at rest compared to those without. In addition, RV systolic function reserve as evaluated by the increase in TAPSE after DSE in normal subjects, was abolished in the presence of resting LV-WMA.

As we found, measurement of TAPSE is feasible and easy to obtain, and the values are similar to those found in a previous study which proved the accuracy of this parameter in assessing RV systolic function compared to other parameters like fractional area change [2]. Another study also found excellent correlation between TAPSE and RV fractional area change [9]. TAPSE predicted survival in patients with pulmonary hypertension [10]. However, TAPSE showed a poor correlation with RV ejection fraction in the presence of severe tricuspid regurgitation [11]. Moreover, RV systolic function is not the sole determinant of TAPSE [12]. Similar to our findings, others reported that TAPSE is affected by LV systolic function, and added significant information to prognosis in patients with heart failure [13, 14]. Thus our TAPSE-findings confirmed those reported previously as detailed above, but in addition we report also the limited increase in TAPSE in the presence of resting LV-WMA.

To answer the question why RV systolic function is affected by LV systolic function abnormalities, understanding the anatomy and relation of the myocardial fibers of both ventricles is essential. Thus, contrary to the left ventricle which is composed of three myocardial fiber layers-superficial obliquely directed fibers, middle circumferentially directed fibers and deep inner layer with longitudinal fibers; the RV wall is composed only of two myofiber layers [15]. The direction of RV myocardial fibers of the superficial layer is circumferential parallel to the atrio-ventricular groove and turns oblique toward the apex, and is continuous with the superficial layer of the LV [16], while the inner myocardial layer of the RV is longitudinally directed from base to apex. Therefore, it seems that the arrangement of myocardial fibers and the continuity between RV and LV myocardial fibers are responsible for the decrease in RV systolic function and reserve, as assessed by TAPSE, in the presence of LV-WMA.

A previous study found that TASV correlated with fractional area change of RV and with TAPSE [9]. Yet, another study found that TASV did not correlate with RV ejection fraction in the presence of severe tricuspid regurgitation [11]. Previously, it was reported that TASV correlated with LV ejection fraction and that 12 cm/sec threshold for TASV identified patients with reduced LV systolic function with high specificity [2]. However, in our study, contrary to TAPSE, TASV was similar in both groups and thus was not affected by the presence of LV-WMA. In addition, we found that despite the increase in TASV after DSE, LV-WMA was associated with less increase in TASV compared to those without WMA. It should be emphasized that TASV was similar at rest and after DSE in the presence or absence of LV-WMA.

Why LV-WMA did not affect TASV significantly and yet it decreased TAPSE? In fact, TAPSE is the integral of velocity (TASV) upon time. Despite similar heart rates in the presence or absence of LV-WMA, the ratio of TAPSE/TASV was higher in the absence of LV-WMA. This time index implying longer contraction time of RV myocardial fibers in the absence of LV-WMA can be explained by higher afterload to RV contraction in the presence of LV-WMA (similar to friction effects with higher deceleration and thus more rapid decrease of velocity). The increase in RV afterload interrupts shortening of RV myocardial fibers prematurely. It seems that regional LV-WMA, by changing geometry may lead to alterations in RV myocardial fibers, and thus may affect contraction period of RV. Increase in pulmonary vascular resistance is another factor which may contribute to these results, however pulmonary hypertension was not found in these patients, and it seems unlikely in this study population. Since TASV was similar in both groups, it can be deduced that LV-WMA did not affect contraction of RV myocardial fibers. Thus, RV contractility seems similar while the afterload impeding contraction of RV myocardial fibers may explain the findings in our study. Since RV size and shape were normal and similar in all subjects, and no significant tricuspid regurgitation was observed, it seems that preload did not affect the results of our study.

The increase in heart rate during DSE studies, significantly shortened effective average TAPSE displacement time. After DSE studies no significant difference in TAPSE/TASV time index was observed between subjects with and without LV-WMA. It seems that the increase in TASV after DSE contributed to the larger TAPSE post DSE in subjects without LV-WMA.

Changes of TAPSE and TASV paralleled changes in MAPSE and MASV, implying the dependence of RV longitudinal systolic function and reserve on the status of LV. There was no significant difference in the ratio of TAPSE/MAPSE between the groups at rest and after DSE. In addition, DSE studies did not change this ratio compared to rest in both groups. The ratio TASV/MASV behaved in a similar fashion to TAPSE/MAPSE ratio, and thus emphasizing the interdependence of RV and LV.

In addition to the interventricular septum and pericardium, the continuity between RV and LV myocardial fibers contributes to ventricular interdependence [9]. Despite the position of the interventricular septum, in this study the values of TAPSE were closer to those of lateral LV-MAPSE compared to septal-MAPSE especially after DSE, as seen in Table 3. However, TASV/MASV was similar at both septal and lateral regions. This means that the period of septal systolic longitudinal motion is shortened, implying higher afterload to septal longitudinal motion compared to the lateral LV wall.

### Limitations

TAPSE and TASV were similar at rest and after DSE in normal subjects and in those with LV-WMA only after DSE. Therefore, in further analysis, both groups were evaluated as one group and were compared to patients with LV-WMA at rest. It seems that, the WMSI which equaled 1 at rest and increased only to 1.12 ± 0.14 after DSE was of small magnitude and therefore the studied RV parameters were normal. It remains to be shown that if WMSI after DSE was higher, the RV parameters would behave abnormally.

### Conclusions

Longitudinal RV systolic performance is related to LV performance, and thus TAPSE is decreased in the presence of LV-WMA. TAPSE increases after DSE studies, however, TPSAE reserve is negligible in the presence of LV-WMA. TASV increases after DSE studies, but was similar in the presence or absence of LV-WMA wall motion abnormality. LV-WMA increases afterload to RV longitudinal systolic performance. Afterload to septal longitudinal motion is higher than to lateral LV and RV systolic motion.
